# Food-Grade Gelatin Nanoparticles: Preparation, Characterization, and Preliminary Application for Stabilizing Pickering Emulsions

**DOI:** 10.3390/foods8100479

**Published:** 2019-10-11

**Authors:** Xin Feng, Hongjie Dai, Liang Ma, Yong Yu, Mi Tang, Yuan Li, Weijie Hu, Tingwei Liu, Yuhao Zhang

**Affiliations:** 1 College of Food Science, Southwest University, Chongqing 400715, China; fx199424@email.swu.edu.cn (X.F.); daihongjie@swu.edu.cn (H.D.); zhyhml@swu.edu.cn (L.M.); yuyong@swu.edu.cn (Y.Y.); teresa6699@email.swu.edu.cn (M.T.); ly2873@email.swu.edu.cn (Y.L.); huweijie01@email.swu.edu.cn (W.H.); tingwei95@email.swu.edu.cn (T.L.); 2 Chongqing Key Laboratory of Soft-Matter Material Chemistry and Function Manufacturing, Chongqing 400715, China; 3 Biological Science Research Center, Southwest University, Chongqing 400715, China

**Keywords:** gelatin, nanoparticles, genipin, food-grade, Pickering emulsion

## Abstract

In this paper, the food-grade gelatin nanoparticles (GNPs) were prepared by a two-step desolvation method and using genipin as a cross-linker. The GNPs with narrow size distribution and good dispersion could be obtained only at pH 12. The effect of the genipin dosage (8–12 wt%) on the GNPs was systematically investigated. The results showed that the cross-linking degree of the GNPs increased with the increasing dosage of genipin, thus leading to a more obvious cross-linking morphology observed from scanning electron microscope (SEM). The obtained GNPs showed a good dispersibility with a size range of 386–438 nm. However, the GNPs cross-linked by 8 wt% genipin dosage revealed a relatively higher size because of the aggregation induced by hydrogen bond. The 10 wt% group had good thermal stability and storage stability. The optical microscopy results showed that the Pickering emulsions (30–50 vol% internal phase) stabilized by the GNPs had good uniformity and stability, even after 30 days of storage time, suggesting that the stable GNPs had great potential in food-grade Pickering emulsions.

## 1. Introduction

Emulsions are colloidal dispersions in which one immiscible liquid is dispersed in another immiscible liquid in the form of small droplets [1]. There are usually three types of emulsion, including oil in water (O/W), water in oil (W/O), and mixed system (water in oil in water (W/O/W) or oil in water in oil (O/W/O)) [2]. Emulsions are extensively used in food, pharmaceutical, cosmetics, and other fields because of its diversity of types [3,4]. However, further applications of the emulsions are limited because of poor stability and the use of surfactants. At this time, Pickering emulsions stabilized by the solid particles, with the advantages of no surfactants, high stabilities, and low cost, have garnered exponentially increasing interest in recent years [5,6]. The stability mechanism of Pickering emulsion is mainly because of the limited coalescence, bridging of droplets by a monolayer of particles, and the formation of gel network or greater steric hindrance to inhibit flocculation, coalescence or Ostwald ripening of the emulsion [7]. Recently, a lot of researchers have focused on the study of Pickering emulsions, which is influenced by pH, particles size, particles concentration, wettability, interface properties of particles, and internal phase volume [8,9]. Furthermore, most of the particles for stabilizing Pickering emulsion are inorganic particles [10], chitin nanocrystal particles [11], protein [12], starch granules [13], and cellulose particles [14].

Compared with other particles, the protein particles have good emulsifying properties and great potential to form soft particles, which is more conducive to the deformation of the particles at the interface to stabilize Pickering emulsion [15]. Generally, the protein particles are mainly prepared by heat induction and anti-solvent precipitation. Liu et al. [16] prepared soybean protein nanoparticles by heat-induced method, and the internal structure of nanoparticles was mainly maintained by hydrophobic interactions and disulfide bonding. Folter et al. [17] found that the zein colloidal particles through an anti-solvent precipitation procedure are influenced by particles concentration, pH and ionic strength. The zein colloidal particles showed a good property in stabilizing O/W Pickering emulsions. Besides the plant proteins, the animal proteins are gradually being studied. The whey protein microgel particles are new food-grade particles to stabilize Pickering emulsions, and the adsorption efficiency of the particles strongly depends on the particle charge or the ionic strength of the aqueous phase [18]. 

Gelatin is the denaturation product of collagen under the action of acid, alkali, enzyme, or high temperature. It mainly comes from animal skin, bone, tendon, and other by-products of animal food processing [19]. Gelatin is suitable for the preparation of Pickering soft particles because of its good gelling properties theoretically. Although gelatin is cheap and easy to obtain, it is difficult to obtain gelatin nanoparticles to stabilize Pickering emulsion owing to its strong hydrophilicity and thermal dissolution [20]. Therefore, there are only few reports on gelatin nanoparticles and its application in the stabilization of Pickering emulsions. Tan et al. [21,22] reported that the gelatin nanoparticles can be prepared by glutaraldehyde as a cross-linking agent and acetone as an anti-solvent. However, the use of acetone and glutaraldehyde restrict its green application in the food field. 

Genipin is a natural covalent cross-linking agent hydrolyzed from natural gardenoside by β-glucosidase. It can interact with free amino groups of proteins to form dark blue pigments used in the fabrication of food dyes. It has good anti-inflammatory and anti-allergic effects, making it more suitable for use as medical materials [23,24]. Most importantly, genipin is about 10,000 times less toxic than glutaraldehyde and has been used in food processing [25,26]. Because of its good biocompatibility, slow biodegradation rate, and high degree of cross-linking, there have been some reports about genipin cross-linked gelatin and its applications, such as in hydrogels [27], scaffolds [28], and drug embedding [29]. Gattazzo et al. [30] prepared the gelatin-based scaffolds using genipin as a cross-linking agent. Because of its high biocompatibility, the prepared scaffolds could be used in engineering of skeletal muscle, supporting future applications of gelatin-genipin biomaterials in the field of skeletal muscle tissue repair. However, there is little available information on the gelatin nanoparticles (GNPs) cross-linked by genipin as well as the GNPs-stabilized Pickering emulsion.

In this study, the GNPs were prepared by a two-step desolvation method using genipin as a cross-linker. The effect of pH and genipin dosage on particles size, contact angle, microstructure, and stability of the GNPs were investigated. The preliminary application of GNPs in stabilizing Pickering emulsion was also studied. The purpose of this study was mainly to provide a green method of GNPs and investigate its potential application for food-grade Pickering emulsions.

## 2. Materials and Methods

### 2.1. Materials 

Gelatin (type B, ~180 g bloom, from porcine skin) was obtained from Sigma-Aldrich (Louis, MO, USA). Genipin (HPLC ≥ 98%) was obtained from Chengdu Conbon Bio-tech Co., Ltd. (Chengdu, China). Soybean oil was purchased from a local supermarket (Chongqing, China). All other chemicals and solvents used in this study were of analytical grade.

### 2.2. Preparation of Gelatin Nanoparticles

The GNPs were prepared by a two-step desolvation method-previously reported by Coester [31] and with some modifications. In brief, 1.25 g of gelatin was dissolved in 25 mL of de-ionized water with stirring at 40 °C. Then, 25 mL of ethanol as a desolvation agent was added to the gelatin solution to precipitate the high-molecular-weight gelatin. The high-molecular-weight gelatin was then re-dissolved in 100 mL of de-ionized water and the pH (8–12) was adjusted with 1 mol/L NaOH solution. Subsequently, the gelatin was desolvated again by dropwise addition of ethanol slowly under constant stirring (magnetic stirrer, HJ-3, Changzhou Guohua Electrical Appliance Co., Ltd., Changzhou, China) until a milky white dispersion was formed. To obtain the cross-linked GNPs, a certain amount of genipin solution (8–12 wt%) was added to this dispersion under stirring at 37 °C for 3 h. Finally, the dispersion was centrifuged at 10,000× *g* (MULTIFUGEX3R, Thermo, Austin, TX, USA) for 20 min, and the precipitation was collected and purified by three times of re-dispersion in ethanol and centrifuging at 20,000× *g*. The GNPs were blown dried by nitrogen and stored at 4 °C for further use.

### 2.3. Fourier Transform Infrared Spectroscopy (FTIR)

The FTIR spectra of GNPs were recorded on an FTIR spectrophotometer (Perkin-Elmer, Boston, MA, USA) using the KBr pressed-pellet method [32]. The measurement was performed in the range of 4000 to 400 cm^−1^ with a resolution of 4 cm^−1^. 

### 2.4. Particle Size Measurement 

Before the determination, the GNPs dispersions were diluted to a concentration of 0.01 wt% with de-ionized water, then particles size and polydispersity index (PDI) of the diluted GNPs dispersion liquid were measured using a Malvern ZEN3690 (Malvern Instruments Ltd., Malvern, UK). All measurements were carried out at 25 °C and performed in triplicate for each value.

### 2.5. ζ- Potential Measurement

The ζ-potential of the gelatin particles was measured with the same Malvern ZEN3690 instrument. The concentration of GNPs dispersions was diluted to 0.01 wt%. All measurements were carried out at 25 °C and performed in triplicate for each value.

### 2.6. Scanning Electron Microscope (SEM)

SEM images were carried out using a scanning electron microscope (HITACHI, Regulus8100, Tokyo, Japan) operating at an acceleration voltage of 5 kV. The solid particles were sputtered with gold for 3 min in an argon atmosphere before observation.

### 2.7. Atomic Force Microscope (AFM)

The morphology of the GNPs under different cross-linkers dosages was imaged by AFM (Ntegra Prima, NT-MDT Spectrum instruments, Moscow, Russia). A drop of the dispersion (1 wt%) was placed on a freshly cleaved mica substrate and then dried for 2 h at room temperature in a desiccator with silica gel. A tapping mode with a Ntegra Prima (Nova, Moscow, Russia) equipped with silicon cantilever probes (5 N/m) was applied for the analysis of the samples in air. The micrographs were analyzed using Nova software.

### 2.8. Wettability

The static three-phase contact angle (θ) measurements were performed using a DSA100 (KRUSS, Hamburg, Germany). The GNPs were prepared as pellets of 15 mm in diameter and 1 mm in thickness, a drop of de-ionized water (5 μL) was then deposited on the surface of the pellets using a high-precision injector. By using a charge couple device (CCD) camera and an image analysis system, the imbibition process was recorded and quantified after 4 min for equilibration. The values of θ are the mean value of three replicates.

### 2.9. Preparation of Pickering Emulsions Stabilized by GNPs

The Pickering emulsions were prepared by mixing the GNPs dispersion and soybean oil by mechanical shearing with an Ultra Turrax XHF-DY homogenizer (10 mm head, Ningbo Xinzhi Biotechnology Co., Ltd., Ningbo, China) operating at 13,500 rpm for 1 min. The concentration of GNPS in the dispersions was 1 wt%. The volume of the oil phase was varied from 30 to 70 vol%. Moreover, all emulsions were stored at rest for 30 days (at 4 °C).

### 2.10. Emulsion Type

The emulsion type was inferred by observing the dispersion behavior of a drop of emulsion in de-ionized water. The emulsion type was identified as O/W if the emulsion drop disperses in water otherwise the emulsion type is W/O.

### 2.11. Microscopy and Image Analysis

The photographs of the emulsions were acquired using a Mobile camera to evaluate the macroscopic phase behavior and hence assess their storage stability. To further visualize the microstructure of the emulsions, the emulsion and the 1% SDS solution were first diluted in equal proportions, about 2 μL of the sample was placed on a clean glass cover slip and the microscopy images were acquired using an upright optical microscope (BX53, OLYMPUS, Tokyo, Japan) equipped with 20× objectives. 

### 2.12. Statistical Analysis

All experiments were performed independently at least three times, and the means and standard deviations were calculated from these measurements.

## 3. Results and Discussion

### 3.1. Effect of pH on Gelatin Particles

**Figure 1:**
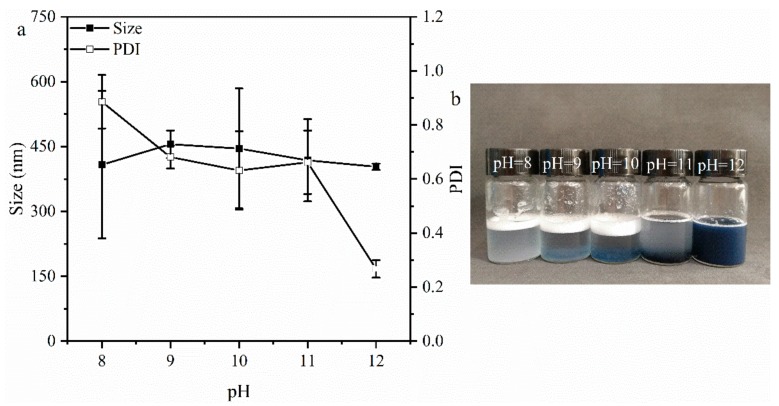
(**a**) Particles size of gelatin nanoparticles (GNPs) prepared at different pH values (pH 8–12). (**b**) Photograph of GNPs dispersions after 1 h of storage (the white layer is the foam layer).

Figure 1a shows the size of the GNPs prepared at different pH values (pH 8–12). At pH 8–11, the size and PDI values of GNPs are 400–450 nm and 0.2–0.9, respectively. The PDI corresponds to the dispersibility of the particles in the solution, the smaller the PDI, the better the dispersion of the particles. Therefore, the GNPs prepared at pH 8–11 are not uniform, corresponding to their poor dispersion based on their PDI values (>0.3). However, at pH 12, the size of GNPs is uniform (about 400 nm), and the value of PDI is about 0.285, implying a good dispersion. At pH 8–11, the electrostatic repulsion was weak, and the drainage capacity of ethanol is great, leading to an irregular aggregation of GNPs. However, the strong electrostatic repulsion inhibits the drainage capacity of ethanol at pH 12, thus obtaining a good dispersion and uniformity of GNPs.

The photograph of GNP dispersions stored at rest for 1 h is shown in Figure 1b. All the dispersions are blue, which is due to the dye formed by cross-linking of genipin with free amino groups in gelatin [33]. Furthermore, there is a little blue precipitation at the bottom of the bottle at pH 8–11, indicating the existence of large aggregates. However, this precipitate has not emerged at pH 12, indicating a good dispersion at this condition. Tan et al. [21,22] used glutaraldehyde cross-linked gelatin to prepare Pickering stabilizer, the suitable pH of gelatin solution was also 12 because of the inhibition of drainage capacity of anti-solvent by the strong electrostatic repulsion between gelatin molecules. Therefore, the subsequent experiments were carried out at pH 12.

### 3.2. Effect of Genipin Dosages on GNPs 

#### 3.2.1. FTIR Analysis

**Figure 2:**
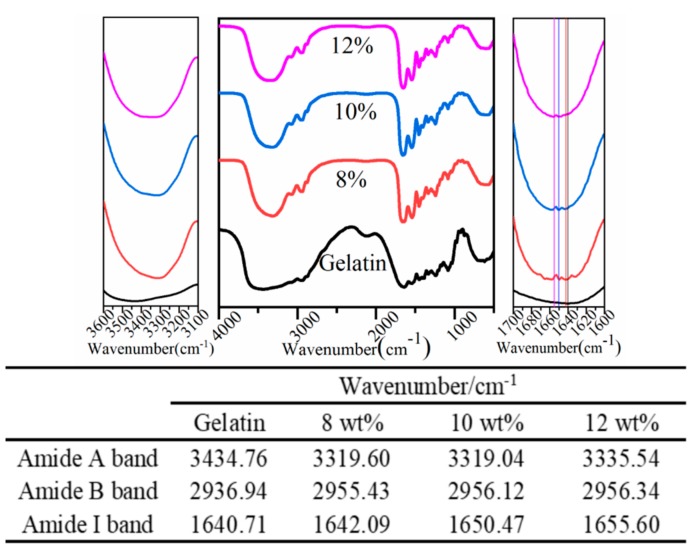
FTIR of GNPs cross-linked by different genipin dosages (8–12 wt%).

FTIR spectra of GNPs cross-linked by different amounts of genipin and the corresponding changes in wavenumbers are shown in Figure 2. In the spectra of native gelatin, a broad peak at 3434 cm^−1^ is attributed to O–H stretching or N–H stretching of amide groups of gelatin, which is a characteristic adsorption of amide A band [34]. The peak at 2936 cm^−1^ is assigned as a characteristic amide B band of protein that results from asymmetric stretching of C–H groups of gelatin [35]. The peak at 1634 cm^−1^ may be attributed to C=O stretching vibration of the amide I band [36].

Compared with the native gelatin, the peak of amide A band of the prepared GNPs shifts to a lower wavenumber with the increase of genipin dosage (8–12 wt%, the three kinds of particles are represented by the 8 wt% group, 10 wt% group, and 12 wt% group, respectively), indicating that N–H groups have participated in the formation of hydrogen bonds [37]. However, the 8 wt% group has the highest shift to low wavenumbers, which implied more hydrogen bonds formation than others. The peak of amide B band shifts to high wavenumbers with the increase of genipin dosages, indicating the increase of hydrogen bonds in peptide chains [38,39]. However, there are no significant changes among the three groups. Usually, in the presence of a cross-linking agent, the Schiff base reaction can be represented by the amide I band shift to high wavenumbers [40]. The amide I band of GNPs cross-linked by different dosage (8–12 wt%) shift toward high wavenumbers. Therefore, the GNPs are formed by the production of Schiff base reaction between genipin and amino groups in gelatin. Moreover, the shift of amide I band in GNPs is more obvious with the increase of genipin dosages (8–12 wt%), implying a higher cross-linked degree created by genipin. 

#### 3.2.2. Particles Size and ζ-Potential Analysis

**Figure 3:**
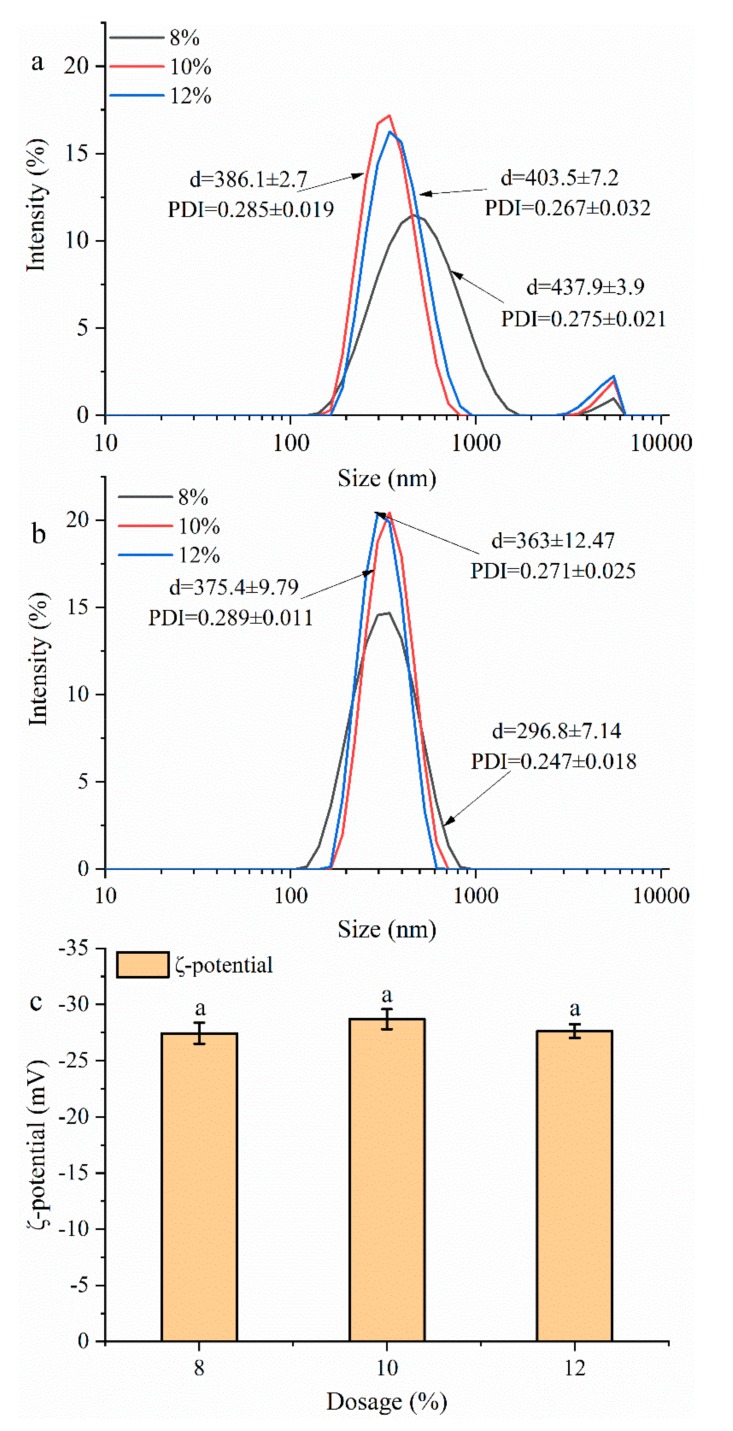
The size of GNPs cross-linked by different genipin dosages (8–12 wt%) that diluted in (**a**) de-ionized water and (**b**) 1% SDS. (**c**) ζ-Potential of GNPs cross-linked by different genipin dosages (8–12 wt%).

As shown in Figure 3a, all three GNPs were relatively uniform and well dispersed. The size of GNPs was not consistent with cross-linking degree, showing a maximum particles size (about 437.9 nm) at the 8 wt% group. This phenomenon may be attributed to the higher degree of aggregation induced by hydrogen bonds (Figure 2), thus leading to an increase in the mean particle size.

To prove this conjecture, the prepared GNPs were dispersed in 1% SDS solution to disrupt the non-covalent bands. As shown in Figure 3b, the sizes of the 8%, 10%, and 12% groups decreased from 437.9, 386.1, and 403.5 nm to 296.8, 375.4, and 360.0 nm, respectively, the more obvious decrease occurred at the 8 wt% group, which is consistent with the degree of cross-linking. ζ-Potential is the most direct characterization of the repulsion or attraction strength between particles, which is related to the stability of colloidal particles dispersion. As shown in Figure 3c, there is no significant difference in the **ζ**-potential of all the three GNPs (*p* > 0.05), which is almost around −30 mV, indicating their high stability.

#### 3.2.3. SEM Analysis

**Figure 4:**
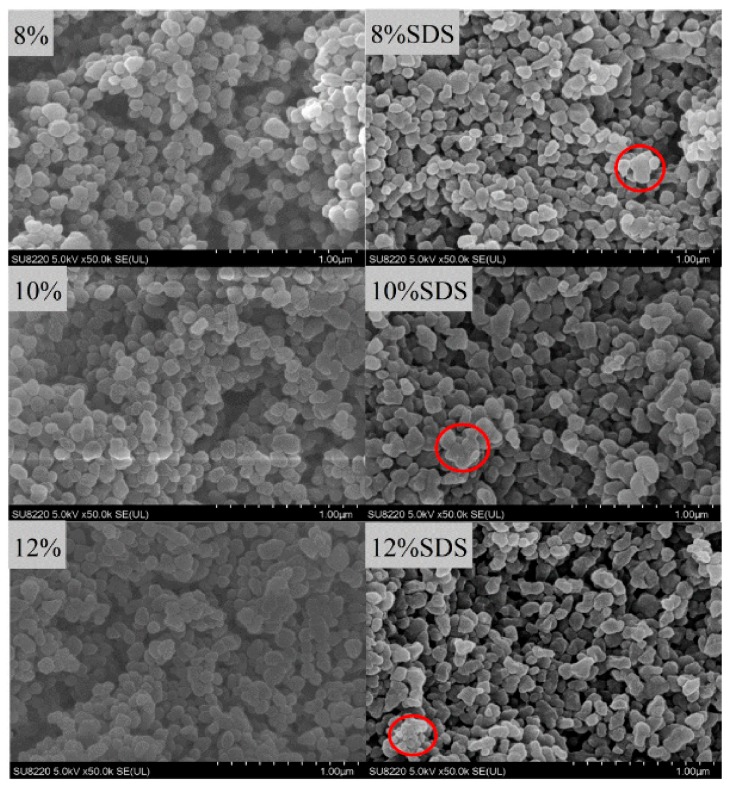
The SEM images of GNPs cross-linked by 8–12 wt% genipin and the corresponding SDS-treated GNPs (the large particles are indicated by a red ring).

The SEM images of GNPs cross-linked by 8–12 wt% genipin and the corresponding SDS-treated GNPs are depicted in Figure 4. As can be seen from Figure 4, all the GNPs are spherical nanoparticles with relatively smooth surface. The size of GNPs is about 100–200 nm, less than the results of particle size distribution by Malvern ZEN3690, which is probably due to the fact that genipin may form intra- and inter-molecular cross-links with cyclic structures in the gelatin chains. A similar phenomenon was also reported by Zhao et al. [41]. In order to prove that the formation of large particles is caused by cross-linking among particles, the GNPs were dispersed in 1% SDS solution to disrupt the non-covalent bands. Obviously, the GNPs under SDS treatment appeared as some large irregular particles (indicated by a red ring in Figure 4). According to previous results (Figure 3a,b), the sizes of GNPs decreased and the dispersions have good PDI after SDS treatment, implying that the GNPs are presented in the form of large particles. 

#### 3.2.4. AFM Analysis

**Figure 5:**
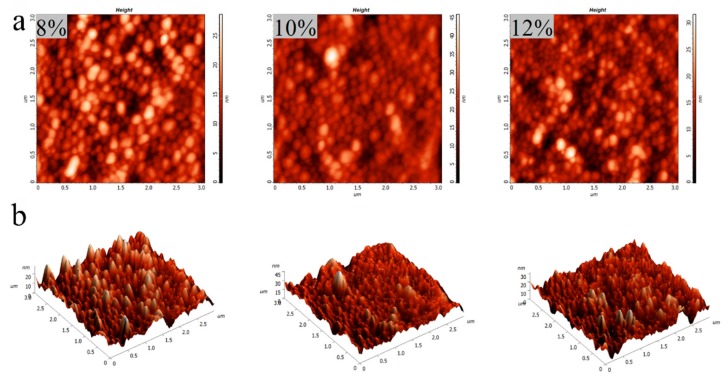
AFM (3 μm × 3 μm) topography (**a**) and three-dimensional (**b**) images of GNPs cross-linked by different genipin dosages (8~12 wt%).

The AFM images of GNPs cross-linked by different genipin dosages are shown in Figure 5. The topography (a) and three-dimensional (b) images show that all GNPs possess a well-defined spherical shape, which is consistent with the observation by SEM. As can be seen from the Figure 5b, 8% group showed the most clusters, indicating that 8% group contained more aggregates, while 10% and 12% group had less aggregates. Moreover, the height of the GNPs is about 12–18 nm, significantly less than the width (100–200 nm), implying the GNPs are soft particles [42,43]. Compared to the rigid particles, the soft ones have some advantages. On the one hand, soft gel particles have a large number of suspending polymer chains on their surfaces that can be anchored at the oil–water interface, as well as biopolymer emulsifiers, which greatly improves their emulsifying ability [44]. On the other hand, after adsorbing onto the interface, the particles can rearrange (deform or flat) at the interface so that a viscoelastic membrane can be formed [45]. Therefore, soft gel particles are more suitable for stabilizing Pickering emulsion than rigid particles. The roughness of the three kinds of particles is about 3 nm, indicating a smooth surface of the obtained GNPs. 

#### 3.2.5. Wettability Analysis

**Figure 6:**
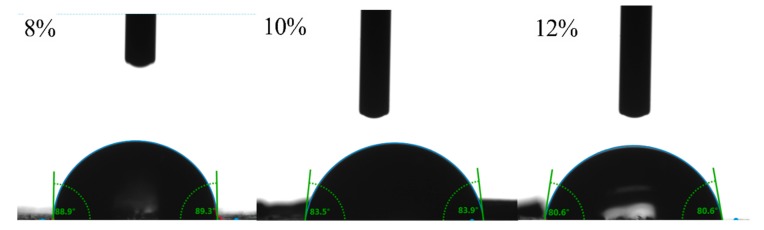
The three-phase contact angle (θ) of GNPs cross-linked by different genipin dosages (8–12 wt%).

The surface wettability of solid particles is an important parameter affecting the type and stability of the emulsion, and the three-phase contact angle (θ) is the most commonly used parameter. According to $E={\pi r}^{2}\gamma {\left(1-\left|cos\theta \right|\right)}^{2}$ (Young’s equation, r is the radius of particles, *θ* is the three-phase contact angle of particles, and *γ* is the interfacial tension between oil and water) [46], the closer the contact angle is to 90°, the larger the energy required for particles to adsorb from the oil–water boundary, thus the obtained emulsion is the most stable. When the contact angle is less than 90°, the particles have stronger hydrophilicity, and are suitable for the preparation of O/W Pickering emulsions. Conversely, when the contact angle is greater than 90°, the particles have stronger hydrophobicity and are suitable for stabilizing the W/O Pickering emulsion. As shown in Figure 6, the θ value of GNPs cross-linked by different genipin dosages (8–12 wt%) decreased from 89.5° ± 0.5° to 81.4° ± 0.8°, indicating that the obtained GNPs are suitable for the preparation of Pickering emulsion. The θ of GNPs are all less than 90°, implying the GNPs are closer to the water phase than the oil phase. Białopiotrowicz et al. [47] have found that the three-phase contact angle of the gelatin film was −65°. It can be seen that the formation of GNPs cross-linked by genipin can significantly change the hydrophilicity of gelatin. With the increase of genipin dosages, the θ value of GNPs decreased, indicating an enhancement of hydrophilicity, which could ascribe that more hydrophobic groups are embedded in the cross-linking process.

#### 3.2.6. Thermal Stability and Storage Stability of Gelatin Particles Analysis

**Figure 7:**
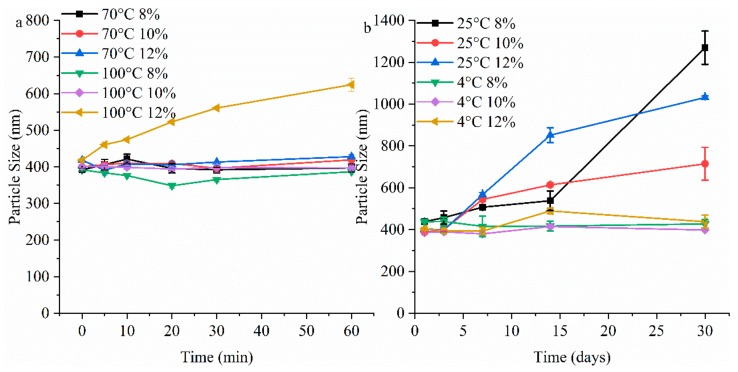
(**a**) The size of GNPs cross-linked by different genipin dosages (8–12 wt%) at different heating temperature (70 and 100 °C) and time (0, 5, 10, 20, 30, and 60 min). (**b**) The size of GNPs cross-linked by different genipin dosages (8–12 wt%) at different storage temperature (25 and 4 °C) and time (1, 3, 7, 14, and 30 days).

Because of the thermal transitions of gelatin, their hydrogen bonds can be destroyed during heating process at above 60 °C, causing melting of gelatin [48]. The sizes of GNPs cross-linked by different genipin dosages (8–12 wt%) at different temperatures and times are shown in Figure 7a, the sizes of all GNPs are almost stable when heated at 70 °C (about 400 nm), indicating that the obtained GNPs have a certain heat resistance. At 100 °C, with the increase of heating time, the sizes of GNPs prepared by the 8 wt% and 10 wt% groups are still stable relatively, implying that covalent cross-linking cannot be destroyed at 100 °C. However, the GNPs prepared by 12 wt% group showed an increase of size with the increase of heating time (*p* < 0.05), which may be due to the Schiff base reaction is strengthened [49]. 

The sizes of GNPs with prolonged storage time are shown in Figure 7b. There is no obvious change in the size (about 400 nm) of the particles during storage at 4 °C, implying their high storage stability under refrigerated conditions. However, at 25 °C, with the increase of storage time, the size of all GNPs increases evidently. After storage for 30 days, the aggregation degree of the 10 wt% group is significantly less than others (*p* < 0.05). This may be due to the fact as follows: the 8 wt% group has more non-covalent aggregated particles, and hydrogen bonds rearrangement may occur gradually during storage, leading to uncontrollable aggregation; the 12 wt% group has the strongest hydrophilicity, and more hydrogen bonds may be formed during storage, which are prone to uncontrollable aggregation, leading to the increase of particles size and decrease of stability.

#### 3.2.7. Gelatin Particles Stabilized Pickering Emulsion

**Figure 8:**
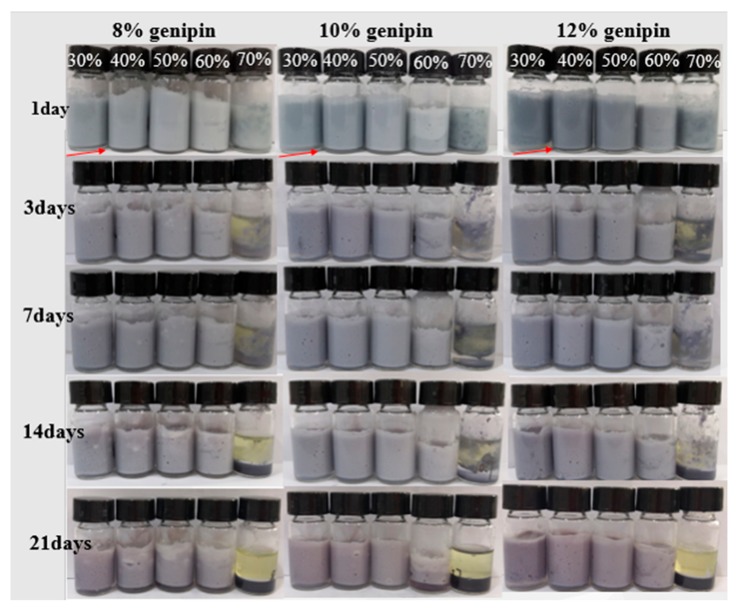
Photograph of Pickering emulsions stabilized by 1 wt% GNPs cross-linked by different genipin dosages (8–12 wt%) at different oil phase volumes (30, 40, 50, 60, and 70 vol%) and storage time at 4 °C (1, 3, 7, 14, and 30 days).

The visual observation and storage stability of Pickering emulsion stabilized by GNPs are shown in Figure 8. As observed from the Figure, all GNPs can form stable Pickering emulsions at oil phase in the range 30–50 vol%. When the oil phase is 30 vol% and 40 vol%, the emulsions show a tiny serum layer at the bottom of the bottles because of creaming. The emulsions display a good uniformity and stability when the inner phase is 50 vol% even after 30 days of storage time. This may be ascribed to the existences of enough particles, which is beneficial for forming gel network or providing more steric hindrance to inhibit flocculation or coalescence [50,51,52]. When the oil phase is 60 vol% and 70 vol%, the emulsions appear highly uneven for macroscopic phase separation. With prolonging the storage time, the emulsion with 70 vol% internal phase appears as an obvious phase separation. This phenomenon could be attributed to the fact that the particles are not enough to cover all the oil droplets completely, leading to phase separation.

**Figure 9:**
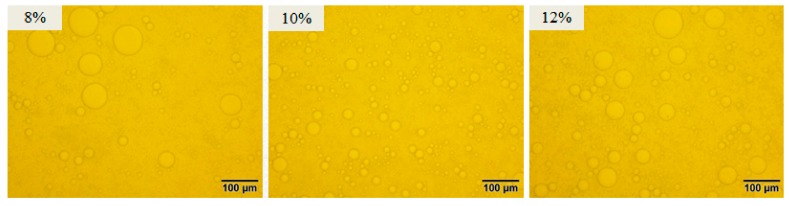
Optical micrographs of emulsions (50 vol% internal phase) stabilized by GNPs cross-linked by different genipin dosages (8–12 wt%).

The result of emulsion type revealed that all the obtained emulsions are oil-in-water (O/W). The optical microscopy of emulsions (50 vol% oil phase) stabilized by GNPs is shown in Figure 9. Obviously, the emulsion stabilized by 10 wt% group showed a higher homogenization than others, which showed that 10 wt% group can make oil droplets disperse better and form emulsions with smaller droplets in the process of emulsification (50% oil phase).

In general, the three kinds of GNPs can be used to prepare a stable Pickering emulsion in the internal phase of 30–50 vol%. Combined with thermal stability and storage stability of GNPs, it is reasonable to assume that the GNPs cross-linked by 10 wt% group is more favorable for stabilizing Pickering emulsions.

## 4. Conclusions

In this study, the green GNPs (about 400 nm) were prepared by using type B gelatin as a raw material and genipin as a cross-linking agent, and used as the food emulsifiers to stabilize O/W Pickering emulsion. The results showed that GNPs with narrow size distribution and good dispersion only could be obtained at pH 12. The GNPs cross-linked by 8 wt% genipin showed a higher particle size (about 437.9 nm) than others, which may be attributed to the existence of a large number of non-covalent aggregates. The SEM images showed the formation of large particles, which is caused by cross-linking among particles. The AFM images showed that all the GNPs appeared as spherical nanoparticles with relatively smooth surface and uniform size. With the increase of genipin dosage (8–12 wt%), the θ value of the obtained GNPs decreased from 89.5° to 81.4°, corresponding to the embedment of more hydrophobic groups in gelatin during the cross-linking process. The stability test results showed that the 10 wt% group possessed a higher thermal stability and storage stability in comparison with others. The storage stability results showed that the GNPs-stabilized Pickering emulsion (30–50 vol% internal phase) showed a higher stability and had no obvious phase separation even after 30 days of storage time. Therefore, the GNPs-stabilized Pickering emulsion can be used in food field owing to the biosafety of GNPs.
